# Novel Use of the GuideLiner Catheter to Deliver Rotational Atherectomy Burrs in Tortuous Vessels

**DOI:** 10.1155/2014/594396

**Published:** 2014-07-23

**Authors:** Minh Vo, Kunal Minhas, Malek Kass, Amir Ravandi

**Affiliations:** Section of Cardiology, Department of Internal Medicine, University of Manitoba and Bergen Cardiac Care Centre, St. Boniface General Hospital, 409 Tache Avenue, Winnipeg, MB, Canada R2H 2A6

## Abstract

Rotational atherectomy (RA) for heavily calcified lesions is essential for improved stent delivery and stent expansion. In tortuous vessels it is often difficult to advance the burr without rotation and possible injury to the endothelium of healthy vessel. The GuideLiner catheter, a child in mother catheter, has recently been used to allow for increased support for delivery of stents through tortuous vessels. We report a novel use of the GuideLiner for the delivery of an RA burr in tortuous vessels requiring increased guide support.

## 1. Introduction

The GuideLiner is a rapid exchange “mother and child” guide extension catheter that allows deep and subselective intubation of the target vessel allowing for improved support during delivery of stents in highly tortuous vessels [1–3]. Rotational atherectomy (RA) which is usually accomplished with the Rotablator burr can facilitate lesion and stent expansion in highly calcific lesions [4]. In many cases due to the tortuosity of the vessel there is an increased risk of vessel perforation and difficulty in delivery of the RA burr to the site of the lesion. We describe 3 cases in which the passage of the GuideLiner beyond the tortuosity within the target vessel allowed delivery of the Rotablator burr enabling safe rotational atherectomy of the calcific lesion. To our knowledge, this is the only reported case of using a Rotablator within a GuideLiner.

## 2. Case Number 1

**Figure 1:**
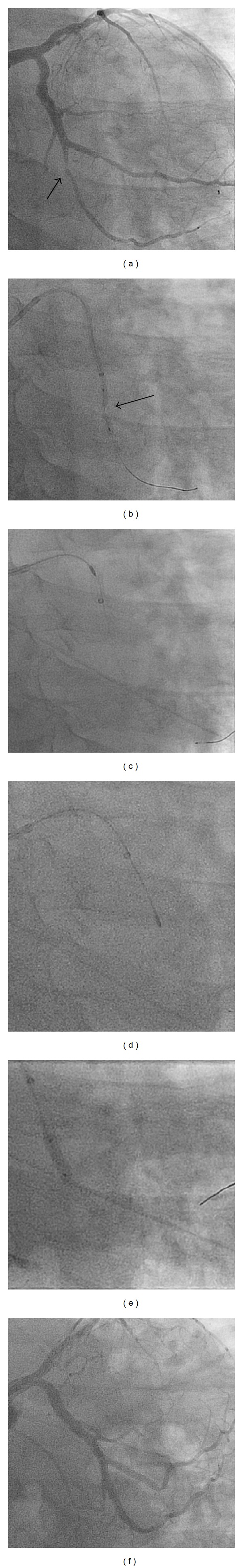
Rotablation of the 2nd obtuse marginal branch of the left circumflex, assisted with the insertion of a 7 Fr. GuideLiner catheter. (a) Severe calcific lesion within OM2 branch (arrow). (b) Resistant lesion within OM2 to noncompliant balloon expansion. (c) Advancement of a 1.25 mm rotablation burr through the GuideLiner. (d) Multiple passes of 1.25 mm burr into the lesion. (e) Successful stent deployment within the lesion. (f) Final results after stent deployment in the OM1 and OM2.

A 67-year-old male with known diabetes, hypertension, dyslipidemia, and previous acute inferior STEMI with previous percutaneous intervention of the RCA and PDA presented with CCS class III angina symptoms refractory to maximal medical therapy. Coronary angiography revealed severe 90% proximal OM2 stenosis and a severe calcific 90% stenosis in the OM3 (Figure 1(a)). The angle of the left circumflex (LCx) artery takeoff from the left main was greater than 90 degrees and its proximal segment had significant calcification as well. Percutaneous coronary intervention (PCI) using the right transradial approach was performed. The initial 6 Fr. sheath was upsized to a 7 Fr. sheath and the left main artery was engaged with an XB 3.5 7 Fr. guide catheter. Unfortunately, a 2.25 noncompliant Quantum balloon (Boston Scientific, Natick, MA, USA) was unable to dilate the calcific lesion at high pressure (Figure 1(b)). A 2.5 balloon was used to dilate and anchor in the midleft circumflex artery to facilitate advancement of the GuideLiner to that position. A 1.25 mm burr was advanced through the GuideLiner and easily negotiated the acute takeoff angle of the left circumflex artery (Figure 1(c)). We then placed the burr just distal to the GuideLiner and performed multiple rotational atherectomy runs at 160,000 rpm (Figure 1(d)). The GuideLiner was then advanced again to the midleft circumflex artery enabling delivery of a 2.5 × 32 Promus Element stent to the proximal OM3. We had good balloon expansion subsequent to rotablation (Figure 1(e)). We treated the OM2 and the proximal LCx with drug eluting stents with excellent final angiographic result (Figure 1(f)). The patient was asymptomatic with no cardiac enzyme elevation the following day and, therefore, was discharged home.

## 3. Case Number 2

This is a 57 y/o male with a history of type 2 diabetes mellitus, hypertension, dyslipidemia, and known coronary artery disease. He had a remote balloon angioplasty to the circumflex. In 2009 he had PCI with Cypher stent to the mid-LAD in setting of myocardial infarction.

**Figure 2:**
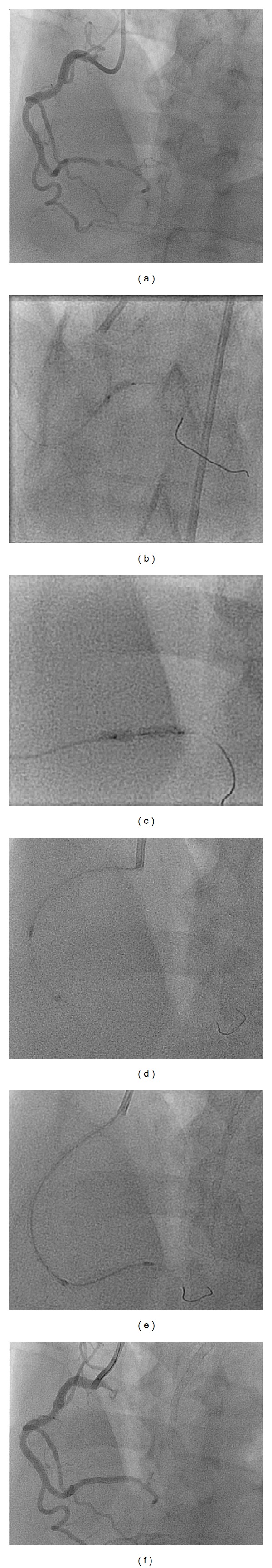
Rotablation of the distal RCA, assisted with the insertion of a 7 Fr. GuideLiner catheter. (a) Tortuous RCA with distal lesion and TIMI 2 flow. ((b) and (c)) Resistant lesion within distal RCA to noncompliant balloon expansion. (d) Advancement of a 1.25 mm rotablation burr through a 7 Fr. GuideLiner. (e) Rotablation of the distal RCA. (f) Final angiographic result after stenting and postdilatation.

The patient presented with ST elevation myocardial infarction in the inferior territory. Cardiac catheterization using right femoral access showed that the left coronary system was unchanged. The distal RCA was subtotally occluded with TIMI 2 flow (Figure 2(a)).

PCI of RCA was undertaken using a 6F AL1 guide and a BMW wire to cross the distal RCA. Three compliant balloons ruptured in the lesion without adequate expansion (Figure 2(b)). The 2.5 mm balloon became stuck in the lesion and on retrieval deep seated the guide catheter resulting in a proximal RCA dissection. The distal RCA lesion was treated with a 2.25 NC Quantum and 2.0 Angiosculpt without expansion. The proximal RCA dissection was treated with a DES. There was final TIMI 3 flow. Patient was admitted to CCU for further decision regarding CABG or another attempt at PCI.

The patient had recurrent angina on IV nitroglycerin and was brought back to the catheterization laboratory the next morning. The plan was for PCI of RCA with GuideLiner support and if required rotational atherectomy. Right radial access with 7F sheath was obtained and a 7F AL1 guide was used to engage the RCA. The lesion was crossed with Pilot 50 wire. We have difficulty advancing noncompliant balloons in the distal vessel due to tortuosity. Next a FineCross microcatheter was used to exchange for an Extra Support RotaWire. A 7F GuideLiner was brought to the distal RCA using balloon inching (Figure 2(d)). A 1.25 mm burr was met with resistance in proximal portion of the GuideLiner. With DynaGlide the burr was delivered to the distal RCA and several passes were made (Figure 2(e)). This allowed for stenting and postdilatation with good final results (Figure 2(f)). The patient was discharged home and has been well on followup.

## 4. Case Number 3

**Figure 3:**
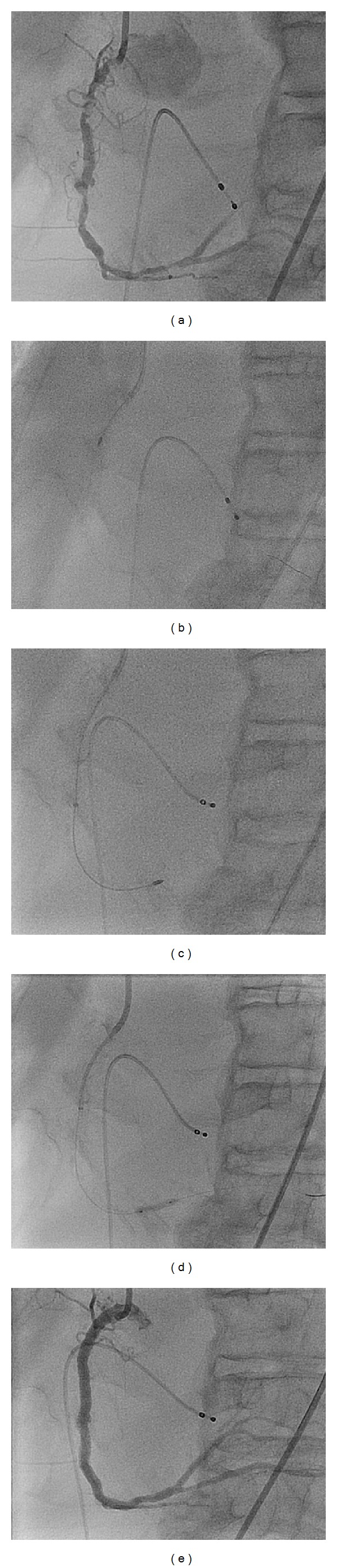
Rotablation of a diffusely calcific tortuous RCA with 7 Fr. GuideLiner. (a) Diffusely calcific RCA showing ostial, mid., and distal severe calcific lesions. (b) Rotablation of the proximal to mid-RCA with difficulty in advancing the Rota. burr in the distal vessel. (c) Advancement of a 1.5 mm burr to the distal RCA through a 7 Fr. GuideLiner. (d) Successful lesion expansion with a noncompliant balloon after rotablation. (e) Final angiographic result with distal to ostial drug eluting stents.

The patient is a 77-year-old male smoker with a history of atrial fibrillation, cerebrovascular disease, diabetes mellitus, and hypertension who presented to the hospital with chest discomfort and a non-ST segment elevation myocardial infarction. He had angiography which demonstrated triple vessel coronary disease with extensive calcification in large dominant right coronary artery (RCA) with EF estimated at 25% with moderate to severe mitral regurgitation. The patient was not deemed a surgical candidate and a percutaneous revascularization strategy was offered. We planned for elective rotablation of the heavily calcified RCA (Figure 3(a)) with intra-aortic balloon pump support given his severe LV dysfunction and mitral regurgitation. Coronary angioplasty was via the right femoral artery with a 7F sheath and access to the RCA with a 7F JR-4 Mach catheter. A whisper wire (Abbott Vascular) was used to cross the lesions in the RCA and, over a FineCross microcatheter, the whisper was exchanged for a RotaWire Extra Support Guide Wire. A 1.25 mm burr was used in the proximal/ostial and midsegments of the RCA (Figure 3(b)). We had difficulty in advancing the burr in the distal vessel due to proximal vessel tortuosity and calcification with poor guide support. The burr was removed in the usual fashion and the RotaWire was replaced using the FineCross with a balance heavy weight (BHW) wire. Predilatation was performed in the proximal and mid-RCA with noncompliant 3.0 × 15 mm balloon at high pressures. We had difficulty in advancing any noncompliant balloons in the distal vessel even with the insertion of a 7 Fr. GuideLiner in the distal vessel. We then switched back to the RotaWire using a FineCross microcatheter with guide trapping technique. Next a 1.5 mm burr was advanced to the proximal segment of the GuideLiner where some resistance was met. With DynaGlide, the burr entered the main body of the GuideLiner and was then advanced in the usual fashion to the tip of the GuideLiner in the mid-RCA. Rotablation was then performed in the distal RCA through the calcific stenosis (Figure 3(c)). The burr was removed and the RotaWire was again replaced with the BHW using the FineCross microcatheter. Final predilatation, stenting, and postdilatation were performed with excellent final results in the RCA (Figure 3(e)). The balloon pump was removed at the end of the procedure, as was the transvenous pacemaker. The patient tolerated the procedure well and was discharged from hospital the following day.

## 5. Discussion

In these series of cases, we describe the novel use of a GuideLiner catheter to facilitate delivery of the Rotablator burr in order to safely and effectively treat a calcific stenosis in a tortuous coronary artery. There has been continuing interest in rotational atherectomy as a tool to treat highly calcific lesion allowing for delivery of balloons and stents and also better stent expansion. As we attempt more complex coronary lesions with improving percutaneous techniques, there is increased use of rotational atherectomy (RA) [5, 6]. One of the main limitations of RA is a stenotic lesion in a very tortuous and angulated coronary artery. In many instances, it is not possible to deliver the burr to the intended target. Furthermore, wire bias can be a source of complication in these complex cases [7]. GuideLiner is a child in mother catheter that is being increasingly used to increase back-up support and to deliver balloons and/or stents to distal coronary lesions [8–10]. However, there has not been a previously reported case of using the GuideLiner to facilitate delivery of Rotablator burr in extremely tortuous and angulated coronary artery. In this report we highlight that we were able to successfully and safely treat a severely calcific lesion in an extremely angulated vessel with the use of RA device delivered through a GuideLiner catheter. The RA burrs used in the present case were 1.25 mm and 1.5 mm burrs. Further experience ex vivo may be useful to check the larger burr sizes which can be safely accommodated through a 7 Fr. GuideLiner catheter. In our experience, in spite of utilizing the DynaGlide mode for burr withdrawal, it is possible that the burr may provide sufficient resistance on withdrawal such that the GuideLiner catheter may “jump” backwards as was the case in our patient. Ex vivo trialing of larger burr sizes through a 7 Fr. GuideLiner would be important to ensure that the burr does not become entrapped in either the distal end of the GuideLiner or even more importantly the proximal metallic transition zone of the catheter. It would be important to ensure the prevention of forward rotational ablation within the GuideLiner catheter whether in ablation or DynaGlide mode to prevent potential damage or shearing of the GuideLiner catheter. It is important to ensure the burr is clearly outside and distal to the GuideLiner catheter prior to initiation of any forward rotation. It is essential to utilize techniques such as these with operators with significant rotational atherectomy experience. Our cases also highlight that in situations with poor guide support the GuideLiner can improve distal delivery of Rota. burr to allow for distal vessel rotational atherectomy.

## 6. Conclusion

GuideLiner and Rotablator are important devices to support complex PCI cases. Each device has different purposes and offers distinctively different advantages. By using both devices simultaneously, we can potentially expand the role of these devices. Severely calcific lesions in distal vessels that are heavily calcified and tortuous can safely and successfully be treated if these two devices are used in unison.
